# Mr. Washbourn's Case of Tic Douloureux

**Published:** 1812-04

**Authors:** N. Washbourn

**Affiliations:** Surgeon, M. R. C. S. Marlborough, Wilts


*To the Editors of the Medical and Physical Journal.*

GENTLEMEN,

If you deem the following case of painful affection of the face *(Tic Douloureux)*, together with a case of Cynanche Trachealis, of sufficient importance for publication, you will oblige me by its insertion in your very useful and interesting Miscellany. I am, Gentlemen,

Your obedient Servant,

*Marlborough, Wilts,* N. WASHBOURN,

*Feb.* 10, 1812. Surgeon, M. R. C. S.

Nov. 8th, 1810. Mr. Lazœron, aged about 50, the Lord Bruce's house steward, made application to me with a most painful and excruciating affection in his face, gums, and head, which he attributed to a number of carious teeth, two or three of which I immediately removed, and which, from the scarification and bleeding, procured a temporary alleviation of his sufferings, which were intolerable. On the 15th I visited him again at his lordship's seat near this town, and found him still suffering as on my last visit. I considered the case to be of the nervous kind, and prescribed medicines accordingly. 19th. Still complaining of the same pain, with little or no mitigation of his sufferings. There being a strong action of the heart and arteries, I was induced duced to diminish the circulating fluid, by abstraction of blood to the quantity of ℥xviij. from the arm, topical application of leeches to the gums, and blisters, and to continue his nervous medicines with the addition of the liquor. Arsenicalis vj. gtt. ter in die. 21st. Extracted several more stumps, in the whole I believe about eight, which he still thought as well as myself were the primary and proximate cause of this painful affection, which still continued, but not quite so violent. He had not taken the medicine so regularly as I could wish, consequently I was disappointed in my expectation of the good effects to be derived from the use of the arsenical preparation. 23d. Attended him again, and found him extremely slow towards a state of convalescence; and his lordship expressed a wish that he would take his medicines more regularly. I adopted the following draughts, with strict injunctions for his taking them properly.

℞. Liquoris Arsenicalis gtt. x. vel xij.
Tinct. Croci. ʒſs.
Aquæ Menth. Virid. ℥j. ft.
Haustus 4tis. horis sumendus.

28th. I visited him again, and found him considerably better; pain in the face nearly gone; had taken the medicine very regularly. Repet. Haust. sumat. j. 6tis. horis. Dec. 2d. Found him quite well.

---

*Case of Cynanche Trachealis, successfully treated with Mercury conjoined with the Mineral Solution.*

May 10th, 1810. I was requested to see a child of Mr. Andrew Greenaway's, about three years of age, laboring under the croup. I instantly adopted a prompt mode of treatment, by first evacuating the stomach, succeeded by the following powders and mixture.

℞. Calomel Hydrosublimat. grs. x.
Sacc. Alb. ℈j. Hydr. Sulph. Rub. grs. vj. trite bene simul et divide in Chart vj. capt. j. 4tis. vel 6tis horis superbibendo Cochl. ampl. j. Misturæ sequentis.

℞. Liquor. Arsenical. gtt. xxx.

Spt. Lavend. Comp. Syr. Tolu. aa ʒj.

Aq. Puræ ℥iij. ft. Mistura.

11th. Visited the child, and found it much better. The stridulous respiration, peculiar to this fatal complaint, much relieved, far beyond my most sanguine expectations. 12th. Visited him again, and found his complaints completely dissipated.

